# A Network Model of the Modulation of γ Oscillations by NMDA Receptors in Cerebral Cortex

**DOI:** 10.1523/ENEURO.0157-23.2023

**Published:** 2023-11-20

**Authors:** Eduarda Susin, Alain Destexhe

**Affiliations:** Institute of Neuroscience (NeuroPSI), Paris-Saclay University, Centre National de la Recherche Scientifique (CNRS), Saclay, France 91400

**Keywords:** γ oscillations, hallucinations, ketamine, network model, NMDA receptors, schizophrenia

## Abstract

Psychotic drugs such as ketamine induce symptoms close to schizophrenia and stimulate the production of γ oscillations, as also seen in patients, but the underlying mechanisms are still unclear. Here, we have used computational models of cortical networks generating γ oscillations, and have integrated the action of drugs such as ketamine to partially block NMDA receptors (NMDARs). The model can reproduce the paradoxical increase of γ oscillations by NMDA receptor antagonists, assuming that antagonists affect NMDA receptors with higher affinity on inhibitory interneurons. We next used the model to compare the responsiveness of the network to external stimuli, and found that when NMDA channels are blocked, an increase of γ power is observed altogether with an increase of network responsiveness. However, this responsiveness increase applies not only to γ states, but also to asynchronous states with no apparent γ. We conclude that NMDA antagonists induce an increased excitability state, which may or may not produce γ oscillations, but the response to external inputs is exacerbated, which may explain phenomena such as altered perception or hallucinations.

## Significance Statement

NMDA synaptic receptors mediate excitatory interactions using the neurotransmitter glutamate. NMDA receptors (NMDARs) have been implicated in psychosis such as schizophrenia and are also targeted by hallucinogenic drugs like ketamine. However, the exact mechanisms of action are still unclear. Furthermore, ketamine paradoxically leads to an excited state, although it is a blocker of NMDA receptors, therefore, in principle diminishing excitation. Here, we use models of cortical networks generating γ oscillations and show that this model can explain the paradoxical exciting effect of ketamine if one assumes a higher affinity on NMDA receptors of inhibitory interneurons. The simulated ketamine effect reproduces known symptoms of psychosis such as increased γ oscillations and exacerbated responses to external inputs, compatible with hallucinations.

## Introduction

Schizophrenia is a mental disorder characterized by three classes of symptoms: positive symptoms (such as delusions, hallucinations and disordered thoughts or speech), negative symptoms (comprehending poverty of speech and deficits of normal emotional response), and cognitive deficits (Lewis et al., 2005; Bozikas and Andreou, 2011; Su et al., 2018). Several abnormalities have been identified in schizophrenic patients, including important differences in neurotransmitters systems, anatomic deficits and abnormal neural rhythms (Uhlhaas and Singer, 2010; Shenton et al., 2001).

γ Oscillations (30–90 Hz) in early-course schizophrenia patients are commonly reported to present increased power and/or phase synchronization (Flynn et al., 2008; Grent-'t-Jong et al., 2018; Perrottelli et al., 2021). In parallel, positive correlation between psychotic symptoms and the γ power have been identified in schizophrenic patients, in which higher γ-band activity corresponded to increased symptom load (Spencer et al., 2004, 2008, 2009; Mulert et al., 2011). These findings indicate that hallucinations and delusions could be related to an excess of oscillatory synchronization in the γ band.

NMDA receptor (NMDAR) antagonists, commonly used in subanesthetic doses as animal and human models to study Schizophrenia (Gunduz-Bruce, 2009), induce a psychotic state that resembles all three classes of symptoms of the disease (Javitt and Zukin, 1991; Krystal et al., 1994; Kalsi et al., 2011). Furthermore, NMDAR antagonists also increase γ power amplitude, both in human and in animal models (Plourde et al., 1997; Pinault, 2008; Hong et al., 2010; Nicolás et al., 2011; Kocsis, 2012; Wood et al., 2012; Shaw et al., 2015; Slovik et al., 2017).

In this study, we investigate by means of computational models how NMDAR antagonists, such as ketamine, affect the dynamics of neural networks and how the generated boosting of γ activity affects the network response, providing an interpretation for the observed correlation between γ power and psychotic episodes.

## Materials and Methods

We describe the model used and the analysis procedures applied to the model.

### Neuronal model

The model consists of a sparsely randomly connected network of excitatory and inhibitory spiking neurons, where the neural units are described by the Adaptive Exponential Integrate-And-Fire Model (AdEx; Brette and Gerstner, 2005). In this model, each neuron *i* is described by its membrane potential $V_{i}$, which evolves according to the following equations:

(1) $$\begin{array}{l} C\frac{dV_{i}(t)}{dt}=-g_{L}(V_{i}-E_{L})+g_{L}\Delta exp\left[\frac{\left(V_{i}(t)-V_{th}\right)}{\Delta }\right]\\ -w_{i}(t)-I_{i}^{Syn}(t)\\ \tau_{w_{i}}\frac{dw_{i}(t)}{dt}=a(V_{i}(t)-E_{L})-w_{i}(t)+b\sum_{j}\delta (t-t_{j}) \end{array},$$

where *C* is the membrane capacitance, $g_{L}$ is the leakage conductance, $E_{L}$ is the leaky membrane potential, $V_{th}$ is the effective threshold, Δ is the threshold slope factor and $I_{i}^{Syn}$(t) is postsynaptic current received by the neuron *i* (see next section). The adaptation current, described by the variable $w_{i}$, increases by an amount *b* every time the neuron *i* emits a spike at times $t_{j}$ and decays exponentially with time scale $\tau_{w}$. The subthreshold adaptation is governed by the parameter *a*.

During the simulations, the equation characterizing the membrane potential $V_{i}$ is numerically integrated until a spike is generated. Formally this happens when $V_{i}$ grows rapidly toward infinity. In practice, the spiking time is defined as the moment in which $V_{i}$ reaches a certain threshold ( $V_{th}$). When $V_{i}$ = $V_{th}$ the membrane potential is reset to $V_{rest}$, which is kept constant until the end of the refractory period $T_{ref}$. After the refractory period the equations start being integrated again.

In the developed network two types of cells were used: regular spiking (RS) excitatory cells and fast spiking (FS) inhibitory cells. The cell specific parameters are indicated in Table 1.

**Table 1:** Specific neuron model parameters

Parameter	RS	FS
** ** $V_{th}$	−40 mV	−47.5 mV
** ** Δ	2 mV	0.5 mV
** ** $T_{ref}$	5 ms	5 ms
** ** $\tau_{w}$	500 ms	500 ms
*a*	4 nS	0 nS
*b*	20 pA	0 pA
*C*	150 pF	150 pF
** ** $g_{L}$	10 nS	10 nS
** ** $E_{L}$	−65 mV	−65 mV
** ** $E_{E}$	0 mV	0 mV
** ** $E_{I}$	−80 mV	−80 mV
** ** $V_{rest}$	−65 mV	−65 mV

### Synaptic models

The postsynaptic current received by each neuron *i* is composed by three components: two excitatory, referred to as to AMPA and NMDA synaptic channels, and one inhibitory, referred to as to $GABA_{A}$ channels.

$$I_{i}^{Syn}(t)=I_{i}^{AMPA}(t)+I_{i}^{GABA_{A}}(t)+I_{i}^{NMDA}(t),$$

in which

(2) $$\begin{array}{l} I_{i}^{AMPA}(t)=G_{i}^{AMPA}(t)(V_{i}(t)-E^{AMPA})\\ I_{i}^{GABA_{A}}(t)=G_{i}^{GABA_{A}}(t)(V_{i}(t)-E^{GABA_{A}})\\ I_{i}^{NMDA}(t)=G_{i}^{NMDA}(t)(V_{i}(t)-E^{NMDA})B(V_{i}(t)) \end{array}.$$

$E^{AMPA}$= 0 mV, $E^{GABA_{A}}$= −80 mV and $E^{NMDA}$= 0 mV are the reversal potentials of AMPA, $GABA_{A}$ and NMDA channels. While the AMPA and $GABA_{A}$-mediated currents are fast, NMDA-mediated currents are slower and voltage dependent (Faber and Korn, 1980; Perouansky and Yaari, 1993; Götz et al., 1997; Bellingham et al., 1998). This voltage dependence, because of magnesium block, is accurately modeled by the phenomenological expression B(V) (Jahr and Stevens, 1990):

(3) $$B(V)=\frac{1}{1+exp(-0.062V).({\left[Mg^{2+}\right]}_{o}/3.57)},$$

where ${\left[Mg^{2+}\right]}_{o}$= 1 mm is the external magnesium concentration (1–2 mm in physiological conditions).

Because of the fast dynamics of *AMPA* and $GABA_{A}$ channels, their synaptic conductances ( $G^{X}$ with X = *AMPA*, $GABA_{A}$) are usually modeled to increase discontinuously by a discrete amount $Q^{X}$, every time a presynaptic neuron spikes at time $t_{k}$, and to subsequently decay exponentially with a decay time constant $\tau_{decay}^{X}$ according to the following equation:

(4) $$\tau_{decay}^{X}\frac{dG^{X}{}_{i}(t)}{dt}=-G^{X}{}_{i}(t)+Q^{X}\sum_{k}\delta (t-t_{k}).$$

In which $\overset{}{\underset{k}{\sum }}$ runs over all the presynaptic spike times. The synaptic time constants used for *AMPA* and $GABA_{A}$ synapses are $\tau_{decay}^{AMPA}$= 1.5 ms and $\tau_{decay}^{GABA_{A}}$= 7.5 ms.

NMDA channels synaptic conductances, $G^{NMDA}$, because of their slow dynamics, are usually modeled as a biexponential function characterized by a rise time constant, $\tau_{rise}^{NMDA}$= 2 ms, and a decay time constant $\tau_{decay}^{NMDA}$= 200 ms, according to the following equation:

(5) $$\begin{array}{l} G_{i}^{NMDA}=Q_{i}^{NMDA}s_{i}{\left(t\right)}^{NMDA}\\ \frac{ds_{i}{\left(t\right)}^{NMDA}}{dt}=-\frac{s_{i}{\left(t\right)}^{NMDA}}{\tau_{decay}^{NMDA}}+\alpha x_{i}(t)(1-s_{i}{\left(t\right)}^{NMDA})\\ \frac{dx_{i}(t)}{dt}=-\frac{x_{i}(t)}{\tau_{rise}^{NMDA}}+\sum_{k}\delta (t-t_{k}) \end{array}.$$

In which $Q_{i}^{NMDA}$ is the synaptic strength of the NMDA synapse toward the neuron *i*, α= 0.5/ms and x(t) is an auxiliary variable. The $\overset{}{\underset{k}{\sum }}$ runs over all the presynaptic spike times. Both, $s{\left(t\right)}^{NMDA}$ and x(t), are adimensional.

**Figure 1.**
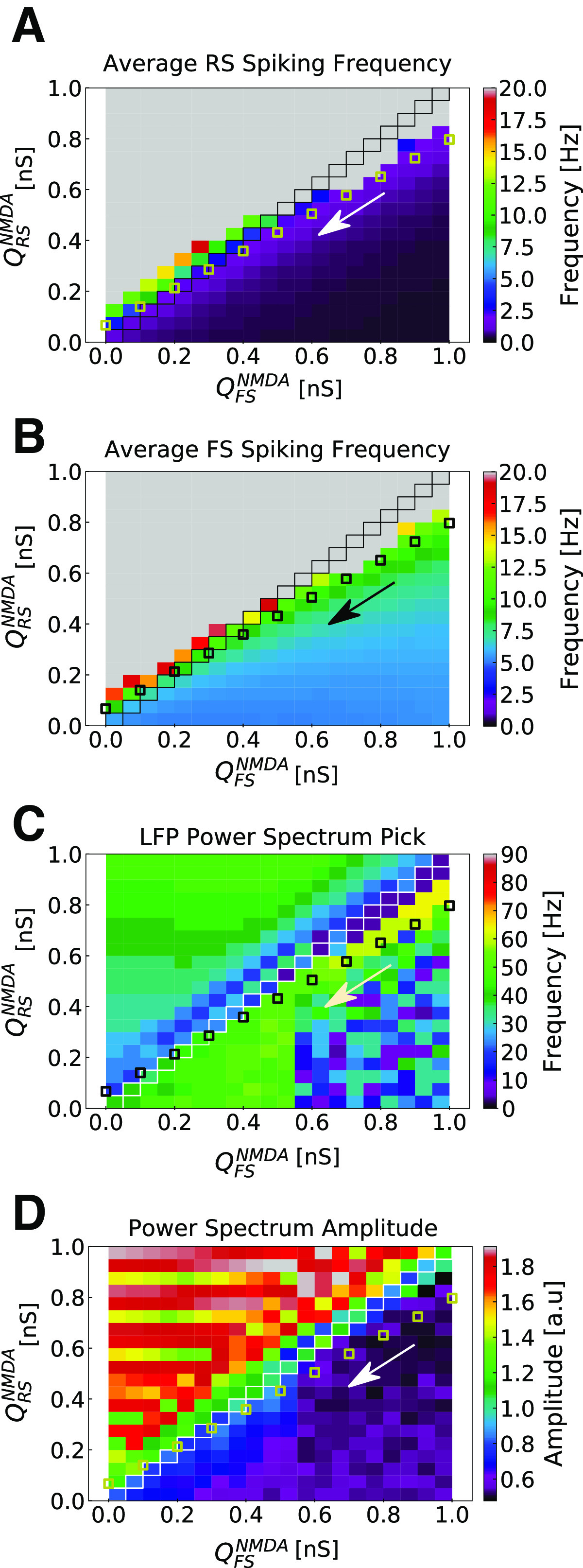
Parameter space of NMDA synaptic weights in RS and FS cells. ***A***, Average spiking rate in RS cells. ***B***, Average spiking rate in FS cells. ***C***, Population activity Power Spectrum pick. ***D***, Population activity Power Spectrum amplitude. The parameter space of weights in NMDA synapses ( $Q^{NMDA}$) was explored for RS and FS cells in the developed network model. $Q_{RS}^{NMDA}$ and $Q_{FS}^{NMDA}$ varied from 0 to 1 nS in steps of 0.05 nS. Each point in the color maps corresponds to the average of 10 simulations of 5 s. Points in which $Q_{RS}^{NMDA}$= $Q_{FS}^{NMDA}$ are highlighted. Small squares indicate a possible trajectory in the parameter space (in the direction of the arrow) generated by the action of NMDAR antagonists.

**Figure 2.**
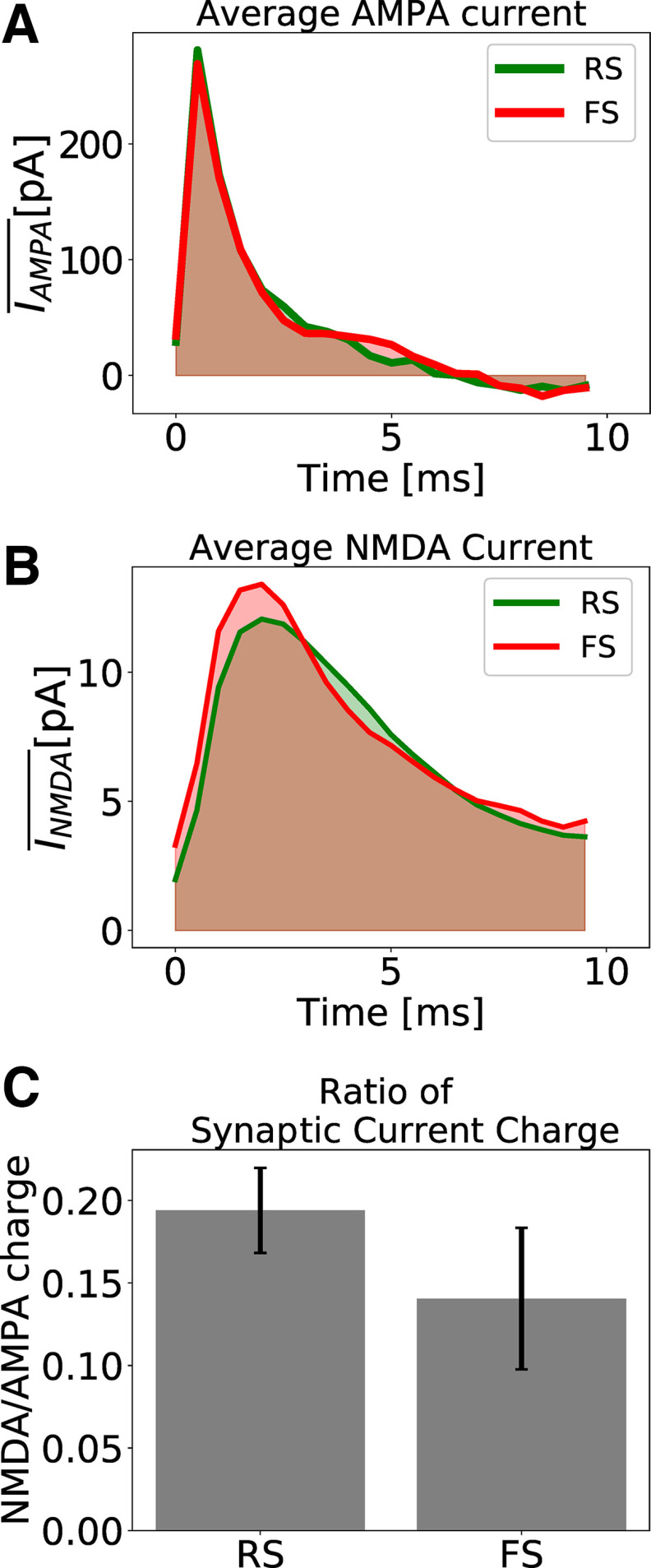
Excitatory synaptic currents. ***A***, Average AMPA current of one randomly picked RS (green) and one randomly picked FS (red) neuron. ***B***, Average NMDA current of one randomly picked RS (green) and one randomly picked FS (red) neuron. ***C***, Ratio of NMDA and AMPA charges for RS and FS cells. The synaptic charge ratio of each neuron was calculated separately. The Bars indicate the mean and the SD among the RS and FS population. The NMDA synaptic strengths in RS and FS cells are $Q_{RS}^{NMDA}$= 0.8 nS and $Q_{FS}^{NMDA}$= 1 nS (which, in our model, describes a healthy condition).

Synaptic strengths of *NMDA* synapses (toward RS and FS neurons) were chosen according to the parameter search expressed in Figure 1 ( $Q_{R}^{NMDA}S$= 0.4 nS and $Q_{F}^{NMDA}S$= 0.5 nS), while the synaptic parameters of *AMPA* and $GABA_{A}$ synapses were chosen according to previous works (Zerlaut and Destexhe, 2017; Susin and Destexhe, 2021; $Q^{AMPA}$= 5 nS and $Q^{GABA_{A}}$= 3.34 nS). All synapses (*AMPA*, $GABA_{A}$, and *NMDA*) were delayed by time of 1.5 ms. With these choice of parameters the NMDA/AMPA charge ratio in the network is on average higher in RS cells then in FS cells (Fig. 2), in agreement with experimental measurements in prefrontal cortex of adult mice (Rotaru et al., 2011) and rat (Wang and Gao, 2009).

### Network structure

The network developed in this work is composed of 5000 neurons (4000 RS and 1000 FS). Each neuron (RS or FS) was connected randomly to every other neuron in the network with a probability of 10%, receiving on average 500 excitatory synapses (mediated by both AMPA and NMDA channels) and 100 inhibitory synapses (mediated by $GABA_{A}$ channels).

### External input

In addition to recurrent connections, each neuron received an external drive to keep the network active. This external drive consisted of $N_{ext}=5000$ independent and identically distributed excitatory Poissonian spike trains with a spiking frequency $\mu_{ext}$. These spike trains were sent to the network with a 10% probability of connection and were computed inside of the synaptic current term $I^{AMPA}$, with a synaptic strength of $Q_{Ext}^{AMPA}$= 0.8 nS. For γ activity, the network was stimulated with a drive with $\mu_{ext}$= 3 Hz. For asynchronous-and-irregular activity, the network was stimulated with a drive with $\mu_{ext}$= 2 Hz. The external drive mimicked cortical input, as if the network were embedded in a much bigger one.

To inspected how the network responded to slowly-varying inputs (occurring in a time window much bigger than the γ period), an additional external input was included in the simulations. A simple way of generation this type of slowly varying stimulus is by means of a Gaussian variation in time of its amplitude. We have chosen Gaussian time variations with SD of 50 ms to allow the stimulus to interact with at least three 60-Hz γ cycles. Several amplitudes were tested. This additional external input, similar to the external drive, also consisted of $N_{ext}=5000$ independent and identically distributed excitatory Poissonian spike trains, connected to the network with a 10% probability. The difference of this additional input was its firing rate time dependence [ $\mu_{ext}(t)$]. These spike trains were computed inside of both synaptic current terms $I^{AMPA}$ and $I^{NMDA}$, with a synaptic strengths of $Q_{Ext}^{AMPA}$= 0.8 nS, and $Q_{Ext^{RS}}^{NMDA}$ and $Q_{Ext^{FS}}^{NMDA}$ as indicated in each case.

### Block of NMDA channels: effect of NMDAR antagonists

**Figure 3.**
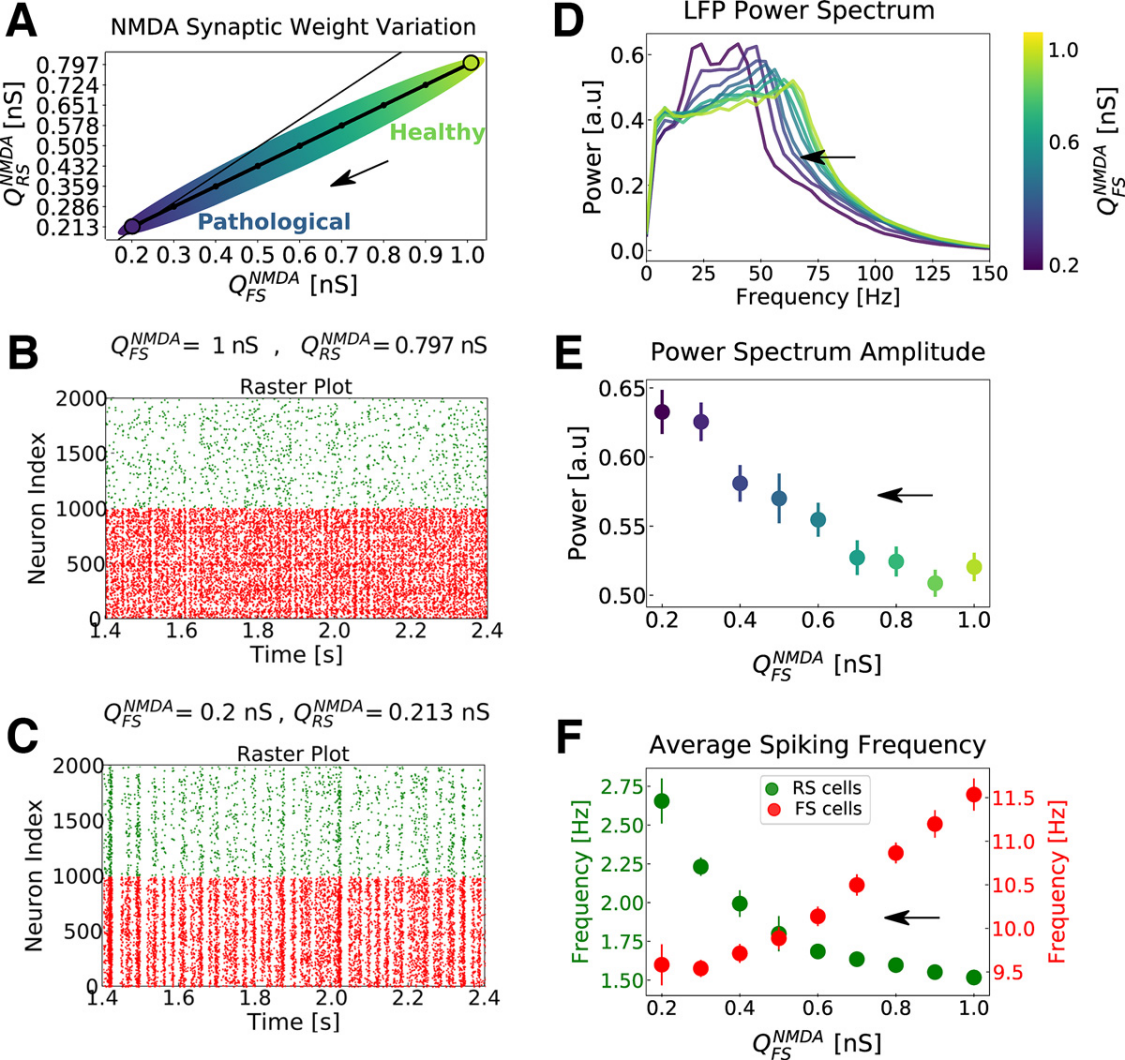
Network dynamics with respect to different levels of NMDA channels block in the network. ***A***, Possible trajectory in the parameter space of $Q_{RS}^{NMDA}$ versus $Q_{FS}^{NMDA}$ (NMDA synaptic strengths in RS and FS cells), mimicking the action of NMDA receptor (NMDAR) antagonists (the higher the intensity of the NMDAR antagonists, the smaller the NMDA synaptic strengths; same trajectory as that indicated in Fig. 1). The thin line indicates the identity for reference. The arrow indicates the progressive action of NMDAR antagonists. Points of higher synaptic strengths are associated with healthy conditions, while points with lower synaptic strengths are associated to pathologic conditions supposedly similar to the schizophrenic brain. ***B***, ***C***, Raster plots indicating the activity of only 1000 cells of each type (FS in red and RS in green), for two parameter sets. ***B***, $Q_{RS}^{NMDA}$= 0.8 nS and $Q_{FS}^{NMDA}$= 1 nS. ***C***, $Q_{RS}^{NMDA}$= 0.213 nS and $Q_{FS}^{NMDA}$= 0.2 nS. ***D***, Average normalized Power Spectrum of the network LFP for different NMDA synaptic strength. The synaptic strengths follow the curve indicated in ***A***, but only the values in FS cells ( $Q_{FS}^{NMDA}$) are indicated in the color scale. Notice the shift of the Power Spectrum peak toward smaller frequencies with the increase of NMDA channel block. ***E***, Power Spectrum peak amplitude with respect to the levels of NMDA channels block. Only the values of the NMDA synaptic strengths in FS cells ( $Q_{FS}^{NMDA}$) are indicated in the *x*-axis. The color scheme (presented for better visualization) is the same as in ***D***. SEMs are indicated as error bars. ***F***, Average firing rate of RS (green) and FS cells (red) with respect to the trajectory in parameter space depicted in ***A***. Like in ***E***, only $Q_{FS}^{NMDA}$ are indicated in the *x*-axis. SEMs are indicated as error bars. Results expressed in ***D–F*** are the outcome of 50 simulations average. In all simulations, an external drive of 3 Hz was used to maintain network activity in γ regime. See Materials and Methods.

In this work, we mimic the effect of NMDAR antagonists by changing the value of the NMDA synaptic weights $Q^{NMDA}$. In Figure 3*A*, a possible trajectory in the parameter space generated by the action of NMDAR antagonists is depicted. This is one of many possible trajectories in parameters space (data not shown).

### Simulations

All neural networks were constructed using Brian2 simulator (Stimberg et al., 2019). All equations were numerically integrated using Euler methods and dt = 0.1 ms as integration time step. The codes for each one of the three developed networks are available at ModelDB platform.

### Population activity: LFP model

To measure the global behavior of the neuronal population, we used a simulated local field potential (LFP). This LFP was generated by the network, by means of a recent method developed by (Telenczuk et al., 2020). This approach calculates the LFP by convolving the spike trains of the network with a kernel that have been previously estimated from unitary LFPs (the LFP generated by a single axon, uLFP) measured experimentally. Since this method assumes a spatial neuronal displacement, to be able to apply it to our simulations, we randomly displaced part of the network (50 neurons) in a 2D grid, assuming that the electrode was displaced on its center and was measuring the LFP in the same layer as neuronal soma. The program code of the kernel method is available in ModelDB (http://modeldb.yale.edu/266508), using python three or the *hoc* language of NEURON.

### Power Spectrum

The Power Spectrum [or Power Spectral Density (PSD)] of the simulated LFP was calculated by means of the Welch’s method, using a Hamming window of length 0.25 s and 125 overlapping points. We used the Python-based ecosystem Scipy function *signal.welch* to do our calculations.

### Distinction between γ and asynchronous states

Because γ oscillations and asynchronous-and-irregular (AI) states are usually part of a continuum of network states (Susin and Destexhe, 2021), we used a criterion based on the PSD to distinguish between γ and AI states. The criterion was that if the oscillatory behavior of the network was prominent, which usually corresponds to a dominant peak at the γ frequency in the PSD, then the network state was considered as γ oscillation. If the PSD peak in the γ frequency was either absent or small compared with the other fluctuations of the PSD, we considered the corresponding network state as AI. It is important to note that for most AI states, although the raster did not reveal any prominent oscillatory component, there was a small peak in the γ frequency range. In general, the amplitude of the oscillation increased with depolarization of the neurons, as described previously (Susin and Destexhe, 2021).

### Synaptic charge

The synaptic charge (AMPA or NMDA) of each neuron is defined as the area under the curve of the average synaptic current (Fig. 2*A*,*B*, shaded areas), which was calculated from the presynaptic input time until 10 ms after it.

### Responsiveness

The level of responsiveness (*R*) of a network, because of an stimulus (*S*) in a time window of duration *T*, is defined as the difference between the total number of spikes generated by the whole network because of an stimulus ( $N_{spikes}^{S}$) and the total number of spikes generated in the absence of the stimulus ( $N_{spikes}$), normalized by the network size (total number of neurons $N_{n}$) and the duration of the time window *T*.

(6) $$R=\frac{N_{spikes}^{S}-N_{spikes}}{TN_{n}}.$$

## Results

We first show that the computational model reproduces features of the block of NMDA receptors observed in cerebral cortex, and next, we investigate the responsiveness of the model in response to external input, comparing γ oscillations with asynchronous states, which will constitute the main prediction of the model.

### Computational model reproduces experimental features

As detailed in Materials and Methods, we used a network model of excitatory RS, and inhibitory FS cells, displaying γ oscillations. This model includes available experimental data on the NMDA/AMPA charge ratio. This ratio is on average higher in RS cells then in FS cells (Fig. 2), in agreement with experimental measurements in prefrontal cortex of adult mice (Rotaru et al., 2011) and rat (Wang and Gao, 2009).

Several preparations with subanesthetics doses of NMDAR antagonists have reported to produce neural excitation (Lahti et al., 1995; Breier et al., 1997; Moghaddam et al., 1997; Vollenweider et al., 1997; Suzuki et al., 2002; Jackson et al., 2004). Since NMDARs mediate excitatory synaptic transmission, this behavior is intriguing. Several hypothesis have been proposed to explain this apparent paradox (Su et al., 2018). One of the possible explanations is that NMDAR antagonists in subanesthetics doses act preferentially on inhibitory neurons, increasing network activity indirectly by means of disinhibition. Although some contrasting results have been observed (Rotaru et al., 2011), this interpretation has been supported experimentally by several works (Lazzaro et al., 2003; Homayoun and Moghaddam, 2007; Zhang et al., 2008; Widman and McMahon, 2018). Network excitability have also been reported to increase in schizophrenic patients (Hoffman and Cavus, 2002; Daskalakis et al., 2007), and its increase in sensory and association cortex have been correlated with hallucinations (Hoffman et al., 2003; Merabet et al., 2003).

Another important effect of NMDAR antagonists in subanesthetics doses is the increase of γ-band activity. These observations were reported in human (Plourde et al., 1997; Hong et al., 2010; Shaw et al., 2015), monkey (Slovik et al., 2017), and rats (Pinault, 2008; Nicolás et al., 2011; Kocsis, 2012; Wood et al., 2012), both during cognitive tasks or free movement.

**Figure 4.**
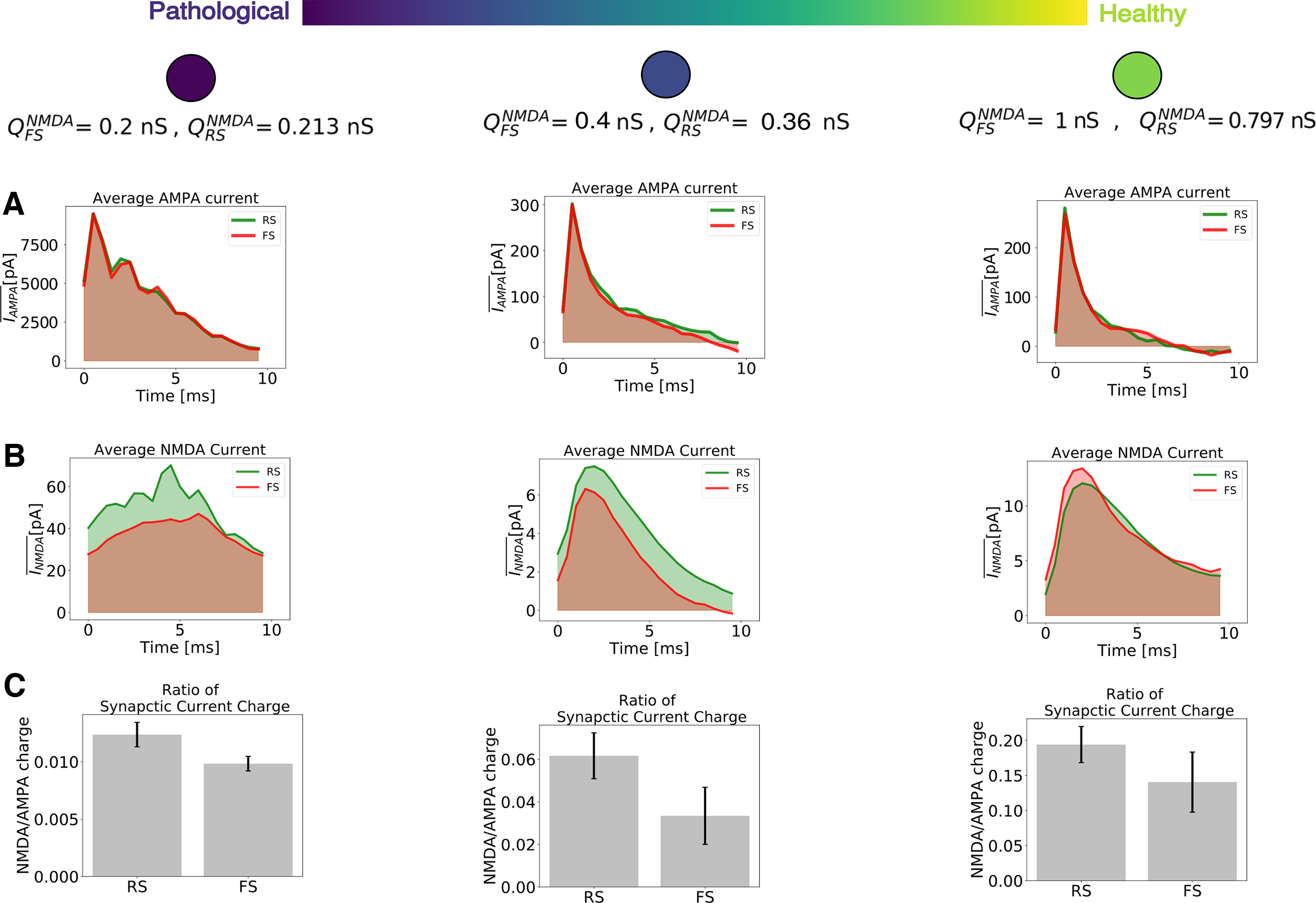
Excitatory synaptic currents with respect to different levels of NMDA channels block. As in Figure 3, higher values of synaptic strengths are associated with healthy conditions, while lower values of synaptic strengths are associated to pathologic conditions supposedly similar to the schizophrenic brain. ***A***, Average AMPA current (for healthy and pathologic conditions) of two randomly picked neurons: RS (green) and FS (red). ***B***, Average NMDA current (for healthy and pathologic conditions) of two randomly picked neurons: RS (green) and FS (red). ***C***, Ratio of NMDA and AMPA charges for RS and FS cells (for healthy and pathologic conditions). The synaptic charge ratio of each neuron was calculated separately. The bars indicate the mean and the SD among the RS and FS population.

The network model developed in the present work (see Materials and Methods) is able to reproduce both of these features (increase of network excitability and increase of γ power). Figure 3 depicts the network behavior with respect to the different NMDA synaptic strengths, $Q^{NMDA}$, in excitatory regular spiking (RS) and in inhibitory fast spiking (FS) cells. We mimic the block of NMDA channels because of the action of NMDAR antagonists by decreasing $Q^{NMDA}$ in RS and FS cells according to Figure 3*A* (see Materials and Methods). Points of higher synaptic strengths are associated with healthy conditions, while points with lower synaptic strengths are associated to pathologic conditions supposedly similar to the schizophrenic brain. The properties of the synaptic input (average current and synaptic current charge), for different levels of NMDA block, are further shown in Figure 4. The network dynamics for two sets of NMDA synaptic strengths are shown in Figure 3*B*,*C* by means of a raster plot. As the synaptic strengths of NMDA channels decreased (higher concentration of NMDAR antagonists), the firing rate of excitatory RS cells increased while the firing rate of inhibitory FS cells decreased (Fig. 3*D*). In addition, the γ power of the population activity (see Materials and Methods) presented an increase (Fig. 3*E*,*F*).

### Network responsiveness during γ rhythms in different levels of NMDAR block

We investigated how the decrease of NMDA synaptic strength changed the network dynamics and its capacity to respond to external stimulus.

While network excitability is related to an overall increase of spiking activity, network responsiveness relates to the network capacity to react to a certain stimulus, producing additional spikes then the ones generated by spontaneous activity. These two dynamical measurements (excitability and responsiveness) are not always congruent, meaning that it is possible to observe an increase in excitability but a concomitant decrease in responsiveness (Susin and Destexhe, 2021).

**Figure 5.**
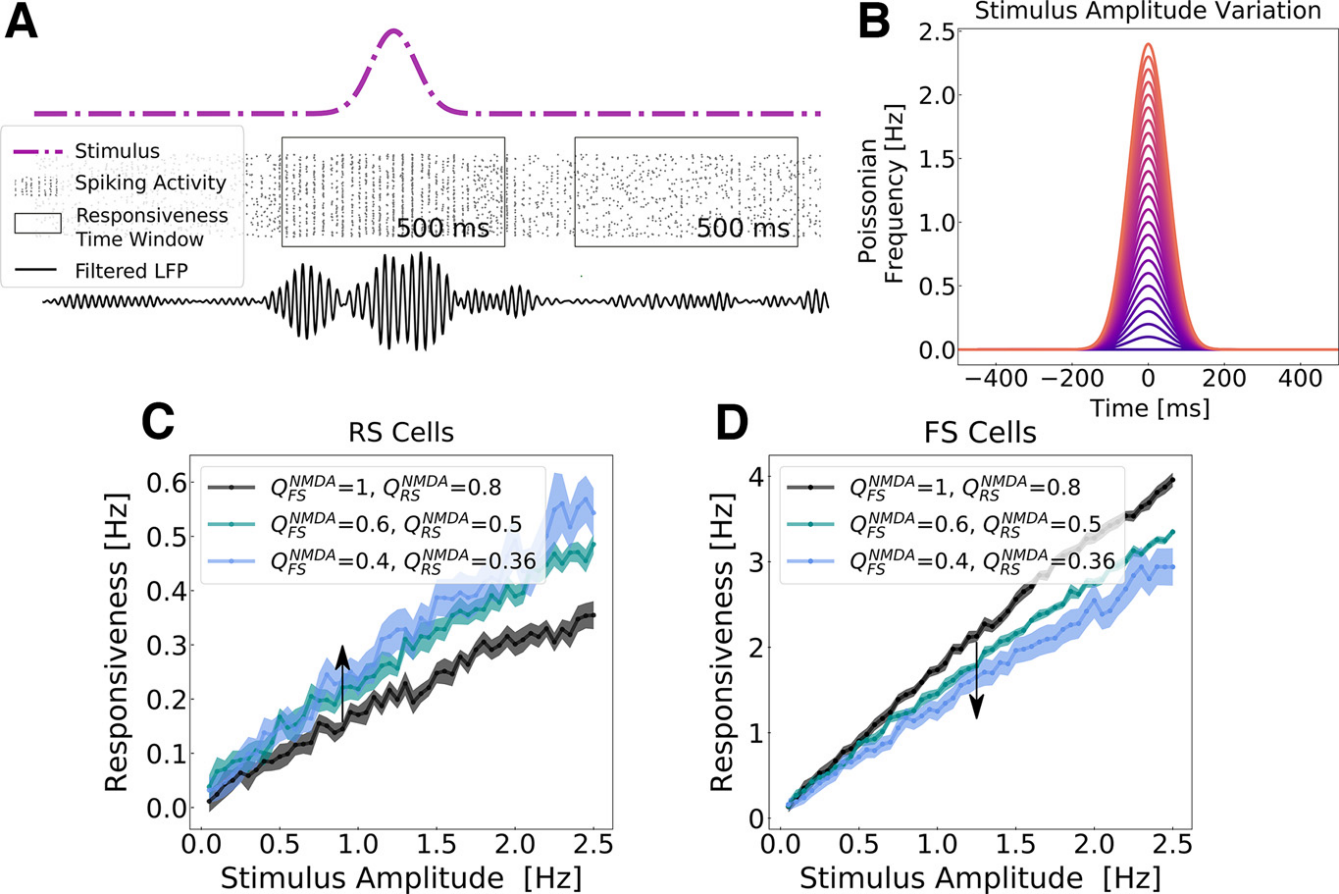
Network responsiveness to broad Gaussian inputs in different levels of NMDA channel blocked during γ rhythms. ***A***, Responsiveness protocol scheme. The total number of spikes generated by the network were measured during an external stimulus and in its absence in a time window of 500 ms. The stimulus consisted of a Gaussian fluctuation in the firing rate of the external noise input. Responsiveness was calculated according to Equation 6. ***B***, Gaussian input amplitude variation. The Gaussian amplitude varied from 0.05 to 2.5 Hz (step of 0.05 Hz), always keeping the same SD of 50 ms. ***C*** and ***D*** depict, respectively, the responsiveness of RS (***C***) and FS (***D***) neurons for different Gaussian amplitudes in different levels of NMDAR block, when the network was displaying γ activity. The color scheme indicates the synaptic weights of NMDA synapses ( $Q^{NMDA}$) in RS and FS cells. The arrow indicates the sense of the simulated action of NMDA antagonist (decreasing synaptic strength). Every point corresponds to the average responsiveness measured in 15 simulations. SEMs are indicated by the shaded region around each curve.

Network responsiveness was defined as the difference between the total number of spikes generated by the whole network in the presence and in the absence of the stimulus (see Eq. 6). We measured network responsiveness at different levels of NMDAR block for different stimulus amplitudes (Fig. 5). The stimulus consisted of a variation in time of the external Poissonian drive in a Gaussian manner (see Materials and Methods).

Network responsiveness in RS cells increased with the increased level of NMDAR block, while the responsiveness of FS neurons decreased. In this case, both, network excitability and network responsiveness, behave in the same direction.

**Figure 6.**
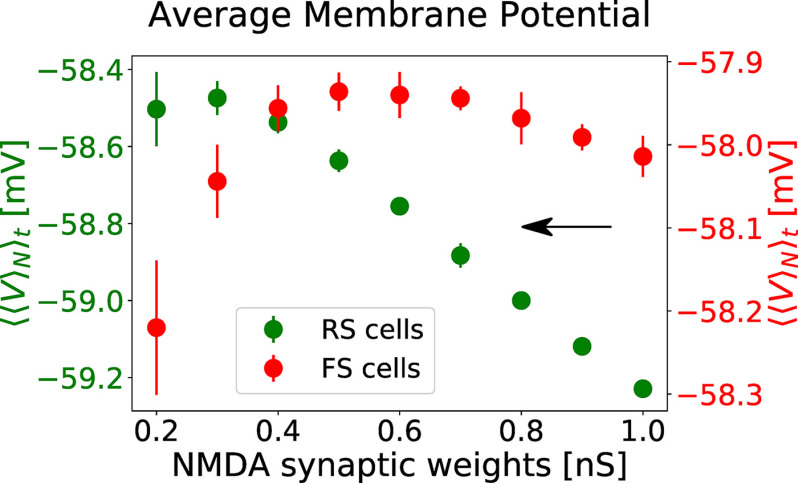
Membrane potential polarization as a function of NMDA receptor block. The average membrane potential of RS (green, left *y*-axis) and FS (red, right *y*-axis) is expressed as function of NMDA synaptic weights of RS and FS cells. The values of $Q_{RS}^{NMDA}$ and $Q_{FS}^{NMDA}$ follow the same trajectory in the parameter space, as indicated in Figure 3*A*. Only the values of $Q_{FS}^{NMDA}$ are indicated in the *x*-axis. The average was performed first in between neurons ( ${〈〉}_{N}$), obtaining an average curve as a function of time, and subsequently with respect time ( ${〈〉}_{t}$). The values plotted correspond to the average of ${〈{〈V〉}_{N}〉}_{t}$ in between 10 simulations. The error bars indicate the SEM between these simulations.

The increase of network responsiveness can be understood from Figure 6. The NMDA receptors block depolarizes RS cells, while FS neurons are overall hyperpolarized. For weak levels of NMDA receptors block, no or weak depolarization is observed in FS cells, while for strong levels of NMDA block a significant hyperpolarization is observed.

### γ States versus AI states

γ Oscillations (30–90 Hz) are believed to be involved in information processing (O’Keefe and Recce, 1993; Singer and Gray, 1995; Singer, 1999; Fries, 2005, 2015; Fries et al., 2007), and have been associated to different high-level cognitive functions, such as memory (Pesaran et al., 2002; Colgin et al., 2009; Carr et al., 2012), perception (Rougeul-Buser et al., 1975; Bouyer et al., 1981; Rodriguez et al., 1999; Melloni et al., 2007), attention (Fries et al., 2001; Gregoriou et al., 2009; Rouhinen et al., 2013; Vinck et al., 2013), focused arousal (Sheer, 1989), and prediction (Womelsdorf et al., 2006). In parallel, studies with schizophrenic patients have reported a positive correlation between psychotic symptoms and the power of γ oscillations (Spencer et al., 2004, 2008, 2009; Mulert et al., 2011).

In contrast, asynchronous-and-irregular (AI) states (Brunel, 2000) are usually associated to conscious states (Koch et al., 2016), being observed during awake and aroused states (Goldman et al., 2019). This regime are characterized by irregular and sustained firing with very weak correlations (Softky and Koch, 1993; Holt et al., 1996; Shadlen and Newsome, 1998; Destexhe et al., 2003; Henrie and Shapley, 2005).

In a previous study (Susin and Destexhe, 2021), we reported that AI states, in comparison to oscillatory states in γ band, provide the highest responsiveness to external stimuli, indicating that γ oscillations tend to overall diminish responsiveness. This observation could indicate that γ rhythms present a masking effect, conveying information in its cycles on spike timing at the expense of decreasing the strength of the network response.

**Figure 7.**
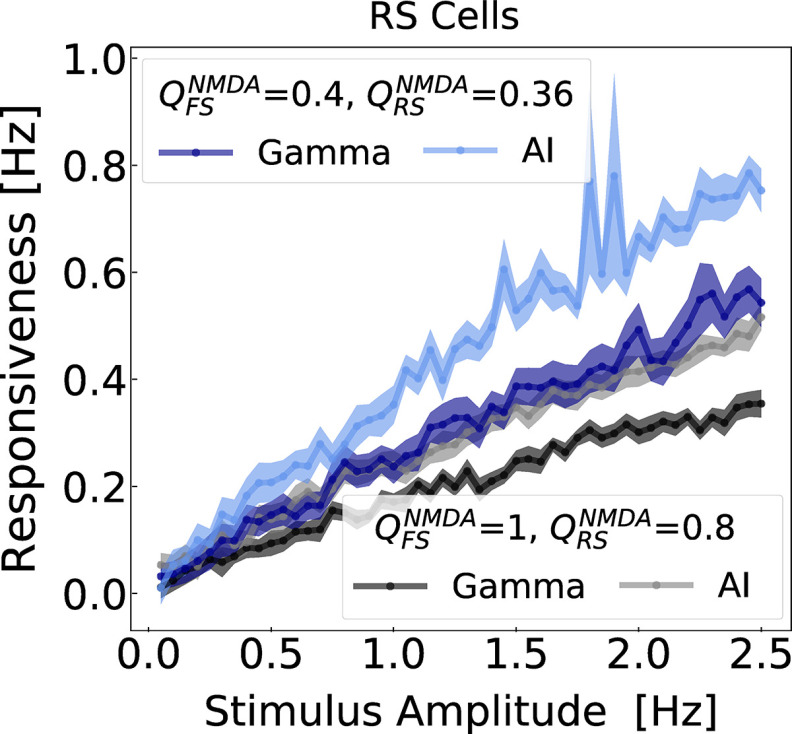
Network responsiveness to broad Gaussian inputs of different amplitudes during γ and AI states. The responsiveness of RS neurons, because of different Gaussian amplitudes stimuli (same as in the protocol of Fig. 5), was measured in different states AI and γ for NMDA synaptic parameter sets: $Q_{RS}^{NMDA}$= 0.8 nS and $Q_{FS}^{NMDA}$= 1 nS (γ: black, AI: gray), and $Q_{RS}^{NMDA}$= 0.36 nS and $Q_{FS}^{NMDA}$= 0.4 nS (γ: blue, AI: light blue). The Gaussian amplitude varied from 0.05 to 2.5 Hz (step of 0.05 Hz), always keeping the same SD of 50 ms.

**Figure 8.**
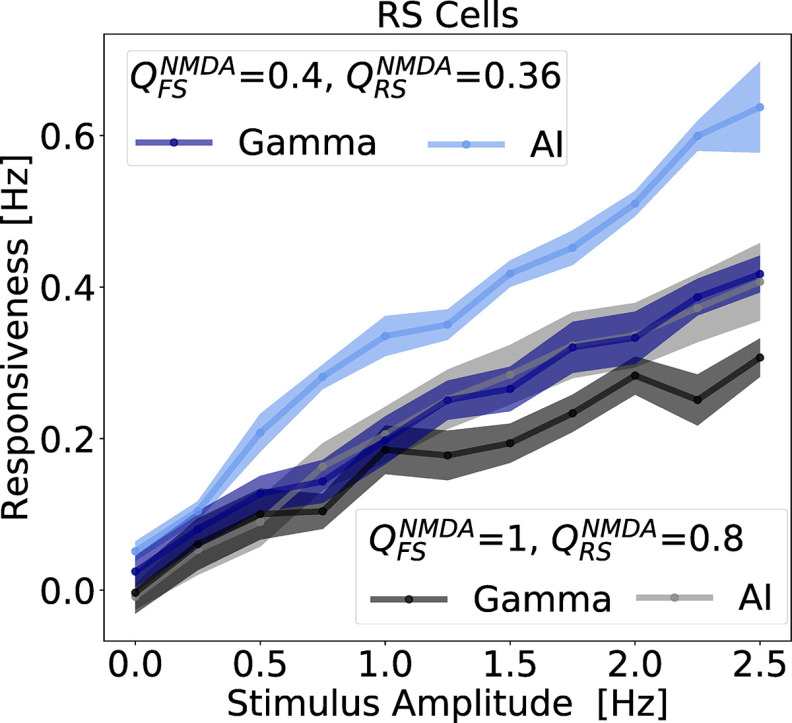
Network responsiveness with faster NMDA decay time constant. As in Figure 7, the network responsiveness to broad Gaussian inputs of different amplitudes during γ and AI states are displayed. In this case, the used NMDA synaptic times constant used in Equation 5 are $\tau_{rise}^{NMDA}$= 2 ms and $\tau_{decay}^{NMDA}$= 50 ms. The responsiveness of RS neurons, was measured in different states AI and γ for NMDA synaptic parameter sets: $Q_{RS}^{NMDA}$= 0.8 nS and $Q_{FS}^{NMDA}$= 1 nS (γ: black, AI: gray), and $Q_{RS}^{NMDA}$= 0.36 nS and $Q_{FS}^{NMDA}$= 0.4 nS (γ: blue, AI: light blue). The Gaussian amplitude varied from 0.25 to 2.5 Hz (step of 0.25 Hz), always keeping the same SD of 50 ms. Every point corresponds to the average responsiveness measured in 10 simulations. SEMs are indicated by the shaded region around each curve.

In the present study, we compare AI and γ states at different levels of NMDAR block. Figure 7 depicts the responsiveness of RS neurons, with respect to different stimulus amplitudes (same protocol as Fig. 5), for different ensembles of NMDA synaptic strengths. In agreement with Figure 5, parameter sets in which NMDA synaptic strengths are decreased (mimicking the action NMDAR antagonists) correspond to regions of the parameter space with higher responsiveness. For example, $Q_{FS}^{NMDA}$= 0.4 nS and $Q_{FS}^{NMDA}$= 0.36 nS displayed higher responsiveness then the networks in which the NMDA synaptic strengths wrere $Q_{FS}^{NMDA}$= 1 nS and $Q_{FS}^{NMDA}$= 0.8 nS. Interestingly, in both conditions, responsiveness in AI states were always superior to the one in γ. This result was also observed in a similar model in the absence of NMDA channels (Susin and Destexhe, 2021). This example illustrates a general tendency, which was also observed with other parameter sets. In particular, it was observed when NMDA currents had a faster decay time, as shown in the example of Figure 8.

## Discussion

In this work, we used computational models to investigate the effect of psychotic drugs such as ketamine in cerebral cortex, and how γ oscillations relate to these effects. Our findings are (1) NMDA receptor antagonists modulate the rhythms produced by a simple network model consisting of two distinct cell types, RS and FS cells, which generate γ oscillations by means of the PING mechanism (Tiesinga and Sejnowski, 2009). This modulation is obtained assuming that the NMDAR block predominantly affects interneurons for low doses of ketamine. (2) The boosted γ oscillations following partial block of NMDA receptors, was accompanied by an increased responsiveness to external inputs. (3) This increase of responsiveness could also be seen for asynchronous states, with no apparent γ. We discuss below the implications of these findings.

A first prediction of the model is that it was necessary that the antagonism affects predominantly NMDAR receptors on interneurons. This feature is supported by a number of observations. Intuitively, if the NMDAR block would occur predominantly on excitatory cells, then it is difficult to see how diminishing excitation could augment the activity and excitability of the network. This long-standing question was resolved recently by finding that indeed, NMDAR antagonists primarily affects NMDA receptors on interneurons. It was observed that the application of Ketamine or MK-801 in subanesthetic doses leads to an increased activity of glutamatergic neurons both in cortex (Moghaddam et al., 1997; Lazzaro et al., 2003) and in hippocampus (Widman and McMahon, 2018), and that this increase of glutamatergic activity is a consequence of the disinhibition of GABAergic neurons (Homayoun and Moghaddam, 2007; Zhang et al., 2008). In addition, it has also been reported in hippocampus that inhibitory neurons are more sensitive to NMDAR antagonists than glutamatergic neurons (Ling and Benardo, 1995b; Grunze et al., 1996). Thus, our model completely supports these findings, and could reproduce the increase of γ power induced by NMDA receptor antagonists. On the other hand, contrasting results also exist. For example, Rotaru et al. (2011) argue that NMDAR have less impact on the activity of inhibitory neurons than on the one of excitatory neurons, since they and other authors observed that NMDAR block depressed large EPSP–spike coupling more strongly in excitatory than in inhibitory neurons (Ling and Benardo, 1995a; Karayannis et al., 2007; Rotaru et al., 2011).

The second finding, which is probably the main finding of our study, is that the network has a marked increased responsiveness under the boosted γ condition. This increased responsiveness could be tested experimentally either *in vitro*, by testing the response of cortical slices with and without application of NMDAR antagonists, or *in vivo*, by monitoring their response following administration of NMDA antagonists.

The third finding is that the increase of responsiveness is not specific to γ oscillations, because it was also present for asynchronous states with no apparent γ. The underlying mechanism is that the antagonism of NMDA receptors produce an overall depolarization of RS cells, and hyperpolarization of FS cells. Consequently, there is an increase of responsiveness of RS cells, with a corresponding decrease for FS cells, as we observed. In this model, the increase of responsiveness is because of the depolarizing effect on RS cells, and are not because of γ oscillations. Indeed, the highest responsiveness was seen for asynchronous states, also in agreement with a previous modeling study (Susin and Destexhe, 2021).

### Possible implications to understand brain pathologies

Our model exhibits several interesting properties that can be related to pathologies. First, the model provides a possible explanation for the symptoms associated to ketamine and others NMDA receptor antagonists, such as hallucinations. The enhanced responsiveness produced by antagonizing NMDA receptors may explain exacerbated responses to sensory stimuli, which may be related to phenomena such as altered perception or hallucinations. Indeed, it is well documented that ketamine produces hallucinations together with a marked increase of γ oscillations (Lee et al., 2003; Hakami et al., 2009; Jones et al., 2012).

Besides hallucinations, the model seems also a priori consistent with the previously reported role for FS neurons in schizophrenia. Postmortem analysis of schizophrenic patient brains have shown a reduced expression of parvalbumin (PV) and GAD67 (Akbarian et al., 1995; Volk et al., 2000; Eyles et al., 2002; Lewis et al., 2005; Akbarian and Huang, 2006; Inan et al., 2013). In parallel, genetic ablation of NMDA receptors in PV-positive interneurons in rodents mimics important behavioral (Korotkova et al., 2010) and phenotypical features of the disease [reduction of GAD67 (Belforte et al., 2010), increase of neuronal excitability (Belforte et al., 2010), and increase of spontaneous γ power (Carlén et al., 2012; Billingslea et al., 2014; Nakao and Nakazawa, 2014)]. These observations support the idea that the hypofunction of NMDA receptors in PV-positive interneurons are especially important in this illness.

However, NMDA receptors are expressed in both GABAergic and glutamatergic neurons (Homayoun and Moghaddam, 2007), and it still remains unclear in which types of cells the NMDA receptor hypofunction causes schizophrenia (Gonzalez-Burgos and Lewis, 2012; Su et al., 2018). Some works reported conflicting results and have questioned the hypothesis that PV-positive Fast Spiking neurons play a role in Schizophrenia (Rotaru et al., 2011; Gonzalez-Burgos and Lewis, 2012).

In our model, the effect of NMDAR antagonists is to increase excitability because of disinhibition, consistent with a number of experimental observations (Lahti et al., 1995; Breier et al., 1997; Moghaddam et al., 1997; Vollenweider et al., 1997; Suzuki et al., 2002; Jackson et al., 2004). This increased excitability is accompanied by a γ power increase, as also found in experiments with ketamine (Plourde et al., 1997; Hong et al., 2010; Shaw et al., 2015) or in schizophrenic patients (Spencer et al., 2004, 2008, 2009; Flynn et al., 2008; Mulert et al., 2011; Grent-'t-Jong et al., 2018; Perrottelli et al., 2021). The model could reproduce all these experimental observations only assuming a larger decrease of the NMDA synaptic strengths in FS cells than in RS cells (Fig. 3*A*). These results support the idea sustained by some authors (Gonzalez-Burgos and Lewis, 2008), that PV-positive Fast Spiking inhibitory neurons play a key role in schizophrenia. Another modeling study also stressed the importance of NMDA channels into FS neurons (Spencer, 2009). Thus, models support the view that the hypofunction of NMDA receptors on FS cells could explain a number of features typical of schizophrenia, such as anomalous responses and boosted γ oscillations.

## Synthesis

Reviewing Editor: Nicholas J. Priebe, University of Texas at Austin Decisions are customarily a result of the Reviewing Editor and the peer reviewers coming together and discussing their recommendations until a consensus is reached. When revisions are invited, a fact-based synthesis statement explaining their decision and outlining what is needed to prepare a revision will be listed below. The following reviewer(s) agreed to reveal their identity: NONE.

SYNTHESIS

Two reviewers evaluated the manuscript “A network model of the modulation of gamma oscillations by NMDA receptors in cerebral cortex” and both reviewers agree that the authors present a model that provides a network-based explanation for how NMDA receptor antagonists causes a paradoxical increase in excitability. The primary assumption of the model is that NMDA receptors in inhibitory neurons is stronger than in excitatory neurons, which has some experimental support. The authors review papers that indirectly support this assumption, as well as those inconsistent with this assumption. The assumption itself though seems surprising at the mechanistic level. Why should the same antagonist have a different effect on NMDAR’s in excitatory and inhibitory cells. A possible reason could be if the subunit composition of NMDAR on excitatory and inhibitory cells are different or even if they are different in a subpopulation of inhibitory cells, as NMDAR antagonists might have a different affinity to these different subunits. If this is the case, then more selective antagonists or even agonists might be used to indirectly validate the model. Overall, the paper provides a clear account and a testable hypothesis to account for this surprising result. While the authors make compelling arguments, the reviewers noted many details that should be clarified in revising your manuscript, if you decide to resubmit it to eNeuro or to another journal.

Detailed Comments:

1) The 200 ms time constant for NMDAR is very slow. It mostly characterizes NR2B which are more prevalent in young animals, but in older animals it is dominated by NR2A which has a time constant of about 50ms. Do the present results depend on this long time scale?

2) What does “The spiking frequency of the spike trains varied in a Gaussian manner, with a standard deviation of 50 ms ...” mean? This seems to be shown in figure 3A. But a clear explanation in the methods is not found.

3) What are the units on the Y axis of figure 1A,B? Why are numbers 50 fold bigger for NMDAR, indicating a much larger integral, but at the same time 1C indicates that the charge is dominated by AMPA.

4) A strong assumption is made that NMDAR antagonists have a higher affinity for NMDAR on inhibitory interneurons. There seems to be some experimental support for this. However, why would this be the case? Are the different types of NMDAR subunits of excitatory vs. inhibitory neurons? A more direct conformation and mechanistic understanding should be discussed.

5) Fig 2A describes the major assumption of the manuscript. It would be clearer if in addition we see two points of this curve in terms of the NMDA/AMPA ratio as in 1C.

6) Is it experimentally true that network responsiveness of FS cells is several times higher than RS cells? (Fig 3C,D). This seems like a large difference and could be a major prediction to validate in general the models’ structure, and parameter regime.

7) The parametrization of X axis of plots like 2D and 4 are confusing, all of them depend on figure 2A. The line in 2A is a linear curve, it can be parametrized, and then the parameter can be used for these plots.

8) I am confused by figure 5. In figure 2 it seems the network is switched from an AI to a Gamma state by changing differentially NMDAR conductance’s. However, in figure 5, for the same levels of NMDAR conductance’s we have both an AI and a Gamma state. Is there another switching mechanism in the model? Is it this caused by a change in the frequency of external stimulation?

In figure 2, was the same external frequency used for both 2B and 2C? Another possibility is that the network can randomly switch between regimes, but this is never shown.

Minor issues:

1. “The model can reproduce the paradoxical increase of gamma oscillations by NMDA-receptor antagonists, assuming that antagonists affect NMDA receptors with higher affinity on inhibitory interneurons.”

This is unclear. Are you writing that antagonists have a higher affinity to the NMDA receptors on synapses from excitatory to inhibitory receptors compared to those from excitatory to excitatory? How is this justified later in any way?

2. “Furthermore, Ketamine paradoxially (paradoxically) leads to and (an) excited state, while it is a blocker of NMDA receptors, therefore in principle diminishing excitation.”

3. “the NMDA-mediated are considerably slower and present a complex relation with respect to the membrane potential” - odd phrasing.

4) desinhibition -> disinhibition

5) synapctic in Figure 1C

6) referent -> referred to as (multiple locations)
